# Etanercept protects rat cardiomyocytes against hypertrophy by regulating inflammatory cytokines secretion and cell apoptosis

**DOI:** 10.1590/1414-431X20175868

**Published:** 2017-05-15

**Authors:** Q. Li, Q. Yu, R. Na, B. Liu

**Affiliations:** 1 Zhejiang Province Hospital of Integrated Traditional Chinese and Western Medicine, Hangzhou, China; 2 Affiliated Zhongshan Hospital of Dalian University, Dalian, China

**Keywords:** Tumor necrosis factor-α inhibitor, Endothelin, Myocardial hypertrophy, Cell apoptosis, Inflammatory response

## Abstract

We aimed to investigate the effect of etanercept, a tumor necrosis factor-α (TNF-α) inhibitor, on rat cardiomyocyte hypertrophy and its underlying mechanism. Primary neonatal rat cardiomyocytes were isolated from Sprague-Dawley rats. The model of rat cardiomyocyte hypertrophy was induced by endothelin, and then treated with different concentrations of etanercept (1, 10, and 50 μM). After treatment, cell counts, viability and cell apoptosis were evaluated. The mRNA levels of myocardial hypertrophy marker genes, including atrial natriuretic factor (ANF), matrix metalloproteinase (MMP)-9 and MMP-13, were detected by qRT-PCR, and the expressions of apoptosis-related proteins (Bcl-2 and Bax) were measured by western blotting. The protein levels of transforming growth factor-β1 (TGF-β1), interleukin (IL)-1β, IL-6, leukemia inhibitory factor (LIF) and cardiotrophin-1 (CT-1) were determined using enzyme linked immunosorbent assay (ELISA) kits. In the present study, TNF-α level in cardiomyocytes with hypertrophy was significantly enhanced (P<0.05). Compared to the model group, cell number and viability were significantly increased and ratio of apoptotic cells was reduced by etanercept (P<0.05, P<0.01, or P<0.001). In addition, etanercept remarkably reduced the mRNA levels of ANF, MMP-9 and MMP-13, inhibited the expression of Bax, and increased the expression of Bcl-2 compared to the model group (P<0.05). ELISA results further showed that etanercept lowered the levels of IL-1β, IL-6, LIF and CT-1 but not TGF-β1 compared to the model group (P<0.05). Etanercept may protect rat cardiomyocytes from hypertrophy by inhibiting inflammatory cytokines secretion and cell apoptosis.

## Introduction

Cardiomyocyte hypertrophy is a common pathological change of progressive cardiac function failure, which can induce increased ventricular stress, declined cardiac function, cardiomyocyte death and tissues fibrosis (1). Cardiomyocyte hypertrophy is considered an adaptive response to preserve cardiac pump function under stress. However, prolonged hypertrophy could cause some adverse effects such as arrhythmias, functional decompensation, cardiac fibrosis or heart failure, and has been proven to be a major independent risk factor for cardiomyocyte hypertrophy (2). Therefore, it is urgent to search for effective therapeutic drugs and methods for cardiomyocyte hypertrophy.

Accumulating evidence has demonstrated the molecular mechanisms of cardiomyocyte hypertrophy and heart failure are associated with various extracellular factors and signaling pathways (3). Previous studies have shown that adaptive cardiomyocyte hypertrophy is related to interleukin-6 (IL-6) cytokines, growth hormone/insulin-like growth factor-1, and biomechanical stretch signaling, as well as the activation of the signal transducer and activator of transcription 3, the phosphatidylinositol 3-kinase/protein kinase B/glycogen synthase kinase 3β cascade, and extracellular signal-regulated kinases (4–7). Activations of these signaling pathways have been proven to increase cardiomyocyte size and induce concentric hypertrophy in adaptive cardiomyocyte hypertrophy (4–7). In maladaptive cardiomyocyte hypertrophy, tumor necrosis factor-α (TNF-α), angiotensin II and catecholamines (5,8), as well as c-Jun N-terminal kinase and p38 are all involved in cardiomyocyte apoptosis and cardiac fibrosis (9).

Etanercept, a human recombinant soluble tumor necrosis factor-α (TNF-α) receptor protein, is considered to be an effective TNF-α inhibitor (10). It can competitively combine with TNF-α and then block the combination of TNF-α and its receptor, resulting in inhibition of TNF-α activity (10). Several studies have demonstrated that etanercept is used to treat TNF-α-related diseases, such as rheumatoid arthritis (11), Crohn’s disease (12), ankylosing spondylitis (13), psoriasis (14), diabetes mellitus (15), Alzheimer disease (16) and cancers (17). Notably, increased level of TNF-α is found in patients with heart failure, and TNF-α has been proven to serve as a prognostic factor of heart failure (18). A previous study has further revealed that overexpressed TNF-α in mice exerts a detrimental role and may promote the occurrence and development of cardiomyocyte hypertrophy and dilated cardiomyopathy (19). Thus, inhibition of TNF-α expression may be beneficial for cardiomyocyte hypertrophy. However, few studies have investigated the role and mechanism of etanercept in cardiomyocyte hypertrophy.

In the present study, we aimed to evaluate treatment with different concentrations of etanercept on cell growth and cell apoptosis in a rat model of cardiomyocyte hypertrophy l. Furthermore, we also analyzed the levels of myocardial hypertrophy marker genes as well as the expressions of inflammatory cytokines.

## Material and Methods

### Isolation and culture of cardiomyocytes

Healthy newborn (approximately 1–3-day old) Sprague-Dawley rats were used to isolate rat cardiomyocytes following previously reported procedures (20). In brief, rats were sacrificed by cervical dislocation and the heart was removed. Then, the cardiac apex was obtained and rinsed twice with pre-cooled phosphate buffered saline (PBS). After being finely chopped, fragments of the cardiac apex tissues were digested with 0.25% trypsin (Gibco, USA) 5-6 times at 37°C. Next, cardiomyocytes were collected by filtration and centrifugation at 8000 *g* for 5 min at 37°C, and maintained in Dulbecco's modified Eagle's medium (DMEM, Gibco) containing 10% fetal bovine serum (FBS, Gibco) and 5′-bromodeoxyuridine (0.1 mM, Sigma, USA). On day 3, in order to remove the growth medium, the cardiomyocytes were incubated with serum-free DMEM for the next experiments.

### Study design

Rat cardiomyocytes were stimulated with 100 nM endothelin (Sigma) for 4 days in serum-free DMEM to induce cardiomyocyte hypertrophy. To evaluate the effect of etanercept on cardiomyocyte hypertrophy, cardiomyocytes were then treated with different concentrations of etanercept (0, 1, 10, and 50 μM, Sigma) for 48 h.

### Cell counts

After treatment with etanercept, cell counts were performed using trypan blue staining. Cells were collected and resuspended in DMEM medium (1 mL), and then cultured with 0.4% trypan blue stain (100 μL, Gibco). Viable cells were counted in 5 randomly selected fields under inverted microscope (Olympus, Japan), by an investigator blind to the treatments. The ratio of viable cells was calculated as the following formula: (total cell number – number of trypan blue) positive cells/total cell number.

### MTT assay

After treatment with etanercept, cell viability was evaluated by 3-(4, 5-dimethylthiazol-2-yl)-2, 5-diphenyltetrazolium bromide (MTT) assay. In brief, 10^3^ cells/well were seeded into a 96-well plate and cultured for 48 h. After 0, 12, 24, 48 h of incubation, 100 μL of MTT solution (0.5 mg/mL, Sigma) was added into each well and the mixture was incubated at 37°C for 4 h. Then, the culture medium was removed and 150 μL of dimethyl sulfoxide (DMSO, Sigma) was added to the cells to dissolve the formazan crystals. Afterwards, the absorbance at 570 nm was analyzed by a microplate reader (Bio-Rad, USA).

### TUNEL assay

After treatment with etanercept, cells were developed with TUNEL stain kit (Thermo, USA) according to manufacturer's recommendations. In brief, cells were fixed in 10% formaldehyde, and then permeabilized with 0.2% Triton X-100. After treatment with equilibration buffer for 10 min, cells were incubated with terminal deoxynucleotidyl transferase reaction mix for 1 h at 37°C. Cells were then immersed in wash buffer for 15 min, stained with DAPI and finally covered in mounting medium. Positive cells (apoptotic cells) were counted in 5 randomly selected fields under an inverted fluorescent microscope (Olympus).

### qRT-PCR

After treatment with etanercept, the total RNA was extracted from cells using 800 μL Trizol reagent (Invitrogen, USA) and high-quality RNA was reversely transcribed into complementary DNA with the 5× All-in-One RT MasterMix (Applied Biological Materials Inc., Canada). Primers for atrial natriuretic factor (ANF), matrix metalloproteinase (MMP)-9, MMP-13 and β-actin are shown in Table 1. PCR amplification was performed with SYBR¯ Premix Ex Taq™ (TaKaRa, Japan). The PCR program was: 95°C for 3 min, 39 cycles of 95°C for 10 s, and 55°C for 30 s, using the ABI StepOne plus Real-time PCR system (Applied Biosystems, USA). The comparative threshold (Ct) cycle method (2^–ΔΔCt^) was used to calculate relative quantification. β-actin was used as the housekeeping gene.

**Table 1.** Primer sequences for specific genes.

Gene	Primer sequence
MMP-9	Forward: 5′-AAGGATGGTCTACTGGCA-3′
	Reverse: 5′-AGAGATTCTCACTGGGGC-3′
MMP-13	Forward: 5′-CCTGGGATTTCCAAAAGAGGT-3′
	Reverse: 5′-TAACACCACAATAAGGAATTT-3′
ANF	Forward: 5′-CCATATTGGAGCAAATCCTG-3′
	Reverse: 5′-CGGCATCTTCTCCTCCAGG-3′
β-actin	Forward: 5′-CCAAGGCCAACCGCGAGAAGATGAC-3′
	Reverse: 5′-AGGGTACATGGTGGTGCCGCCAGAC-3′

MMP: matrix metalloproteinase; ANF: atrial natriuretic factor.

### Western blotting

After treatment with etanercept, the cells were placed on ice for 30 min in lysis buffer (Beyotime Institute of Biotechnology, China). Supernatant was acquired after centrifugation for 20 min at 12,000 *g* at 4°C. A protein sample was separated on SDS-PAGE gel, and then transferred onto PVDF membranes. The membranes were blocked in 5% skim milk at room temperature for 1 h and then incubated at 4°C overnight with goat anti-rat β-actin monoclonal antibody (1:1000, Santa Cruz, USA), goat anti-rat Bcl-2 polyclonal antibody (1:500, Santa Cruz) and goat anti-rat Bax polyclonal antibody (1:500, Santa Cruz). After being washed for three times with PBS, the membranes were incubated with donkey anti-goat IgG (H+L)-HRP (1:5000, Santa Cruz) for 2 h at 25°C. Ultimately, the proteins were detected with enhanced chemiluminescence (ECL, Applygen Technologies Inc., China) and analyzed by densitometry using the Image-J imaging analysis software (NIH, USA).

### Enzyme linked immunosorbent assay (ELISA)

After treatment with endothelin, the cell culture supernatant was collected to detect the concentrations of TNF-α, transforming growth factor-β1 (TGF-β1), IL-1β, IL-6, leukemia inhibitory factor (LIF) and cardiotrophin-1 (CT-1), which were measured by an ELISA kit (RD, USA), following the manufacturer's protocol. A microplate reader (Bio-Rad) was used to read the absorbance at 450 nm.

### Statistical analysis

Each experiment was repeated five times with at least three replicates. SPSS 19.0 statistical analysis software (SPSS Inc., USA) was used to analyze data. Continuous variables are reported as means±SD and analyzed by one-way ANOVA. P<0.05 was considered to be significant.

## Results

### Effect of etanercept on cell growth in endothelin-induced cardiomyocyte hypertrophy

**Figure 1.**
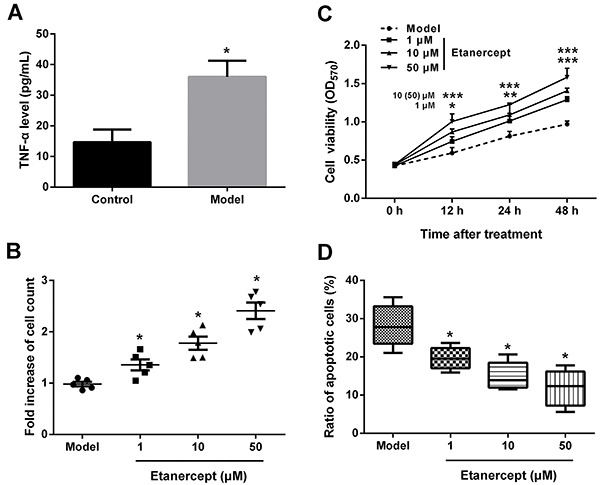
Etanercept promoted cell proliferation but inhibited cell apoptosis in endothelin-induced cardiomyocyte hypertrophy. Cardiomyocytes treated with endothelin alone acted as the model, and non-treated cardiomyocytes acted as the control. *A*, Tumor necrosis factor-α (TNF-α) level assessed by ELISA. *B*, Cell counts assessed by trypan blue staining. *C*, Cell viability assessed by 3-(4,5-dimethylthiazol-2-yl)-2,5-diphenyltetrazolium bromide (MTT) assay. *D*, Ratio of apoptotic cells assessed by TUNEL assay. Data in *A-C* are reported as means±SD. Data in *D* are reported as medians and error bars indicate minimum and maximum values. *P<0.05 compared with control in *A*; *P<0.05, **P<0.01 or ***P<0.001 compared with model (ANOVA).

**Figure 2.**
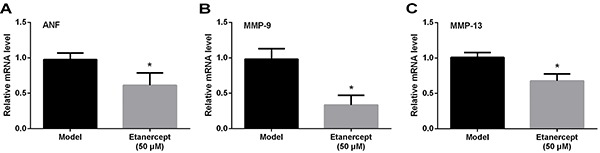
mRNA levels of atrial natriuretic factor (ANF, *A*), matrix metalloproteinase (MMP)-9 (*B*) and MMP-13 (*C*) in cardiomyocytes with endothelin-induced hypertrophy treated with etanercept assessed by qRT-PCR. Data are reported as means±SD. *P<0.05 compared with model (ANOVA).

Compared with non-treated cardiomyocytes (control group), TNF-α level was significantly enhanced in endothelin-treated cardiomyocytes (P<0.05, Figure 1A). Compared with the cell model of endothelin-induced cardiomyocyte hypertrophy, 1, 10, and 50 μM etanercept significantly elevated cell numbers (P<0.05, Figure 1B) and increased cell viability at 12 h (P<0.05 or P<0.001), 24 h (P<0.01 or P<0.001), or 48 h (P<0.001) after stimulation (Figure 2C). In addition, TUNEL results showed that after treatment with different concentrations of etanercept, the ratio of apoptotic cells was markedly lowered than in endothelininduced cardiomyocyte hypertrophy model (P<0.05, Figure 1D). The results also suggested that the optimal concentration of etanercept was 50 μM, and thus, the experiments that followed were performed with 50 μM etanercept.

### Effect of etanercept on myocardial hypertrophy marker genes in endothelin-induced cardiomyocyte hypertrophy

To investigate the role of etanercept in myocardial hypertrophy, the mRNA levels of myocardial hypertrophy marker genes, including ANF, MMP-9 and MMP-13, were detected by qRT-PCR. The results showed that etanercept remarkably reduced the mRNA levels of these markers in comparison with endothelin-induced cardiomyocyte hypertrophy cell model (P<0.05, Figure 2A–C).

### Effect of etanercept on apoptosis-related proteins in endothelin-induced cardiomyocyte hypertrophy

**Figure 3.**
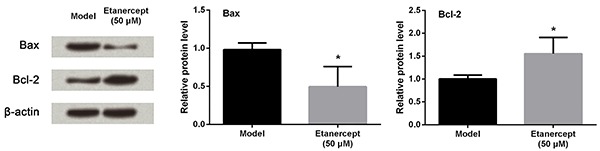
Protein levels of Bcl-2 and Bax in cardiomyocytes with endothelin-induced hypertrophy and then treated with 0 or 50 μM etanercept assessed by western blotting. Data are reported as means±SD. *P<0.05 compared with model (ANOVA).

The protein expression levels of apoptosis-associated proteins were assessed by western blotting. Compared to cardiomyocytes with endothelin-induced hypertrophy, etanercept significantly increased the protein level of anti-apoptotic Bcl-2 and decreased the expression of pro-apoptotic Bax (P<0.05, Figure 3).

### Effect of etanercept on cell inflammatory response in endothelin-induced cardiomyocyte hypertrophy

**Figure 4.**
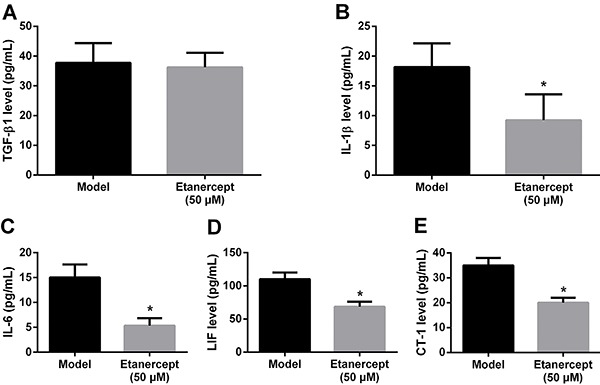
Transforming growth factor-β1 (TGF-β1, *A*), interleukin (IL)-1β (*B*), IL-6 (*C*), leukemia inhibitory factor (LIF, *D*) and cardiotrophin (CT)-1 (*E*) in cardiomyocytes with endothelin-induced hypertrophy and then treated with 0 or 50 μM etanercept evaluated using enzyme linked immunosorbent assay. Data are reported as mean±SD. *P<0.05 compared with model (ANOVA).

To investigate the mechanism of etanercept in rat cardiomyocyte hypertrophy, we detected the levels of inflammatory factors using ELISA. As shown in Figure 4A, there was no significant effect of etanercept on TGF-β1 levels in cardiomyocytes. Conversely, etanercept significantly reduced the levels of IL-1β, IL-6, LIF and CT-1 compared to untreated cardiomyocytes with endothelin-induced hypertrophy (P<0.05; Figure 4B–E).

## Discussion

Although the pathophysiological mechanisms of cardiomyocyte hypertrophy remain unclear, the inflammatory response has proved to be involved in the occurrence and development of cardiomyocyte hypertrophy (21). In the present study, we found that TNF-α level was obviously increased in cardiomyocytes with hypertrophy. Moreover, etanercept significantly promoted cell proliferation and reduced the ratio of apoptotic cells in the cell model of cardiomyocyte hypertrophy. In addition, etanercept remarkably reduced the mRNA levels of myocardial hypertrophy marker genes, inhibited the expression of Bax but increased the expression of Bcl-2. Etanercept also lowered the levels of IL-1β, IL-6, LIF and CT-1, but not TGF-β1 compared with the cell model of cardiomyocyte hypertrophy.

A previous study has demonstrated that endothelin 1 is involved in the development of cardiomyocyte hypertrophy (22), and the cell model of endothelin 1-induced cardiomyocyte hypertrophy had been reported in some experimental studies (23,24). Our study also successfully established the rat cell model of cardiomyocyte hypertrophy by the stimulation of endothelin 1.

ANF is a hormone produced by the heart and mainly serves as a diuretic and hypotensor through inhibiting the secretion of renin and aldosterone (25). ANF is also overexpressed in cardiomyocyte hypertrophy and heart failure. Anti-ANF has been considered an effective method to inhibiting cardiomyocyte hypertrophy by inducing the expression of mitogen-activated protein kinase phosphatase-1 (26). In addition, cardiomyocyte hypertrophy is closely associated with ventricular remodeling and cardiac inflammation, which could contribute to heart failure (27). The production and release of MMPs play an important role during the ventricular remodeling, and MMP-9 and MMP-13 were overexpressed during the progression of remodeling (28). Our study showed that etanercept remarkably reduced the mRNA levels of ANF, MMP-9 and MMP-13, which suggest that etanercept may have an inhibiting effect on cardiomyocyte hypertrophy.

*In vitro* research has suggested that TNF-α could induce down-regulation of sarcomeric proteins, apoptosis and depression of contractility in cardiomyocytes (29,30), indicating that it might play a key role in cardiomyocyte hypertrophy and heart failure. Therefore, the clearance of TNF-α might be a potential therapeutic strategy to improve cardiomyocyte hypertrophy and heart failure. Consistently, we used endothelin to induce cardiomyocyte hypertrophy and not surprisingly found the TNF-α level was markedly increased. Etanercept, a well-known TNF-α inhibitor, is reported to play a pro-apoptotic role in many cells. Malaviya et al. (31) demonstrated that etanercept could induce dermal dendritic cell apoptosis in psoriatic plaques of responding patients. Fries et al. (32) showed the pro-apoptotic role of etanercept in experimental colitis. Avramidis et al. (33) also suggested that etanercept induced apoptosis and reduced apoptosis-inhibiting factors in psoriatic endothelial cells. Similarly, the present study demonstrated that etanercept reduced the ratio of apoptotic cells in cardiomyocytes with hypertrophy. It is well-known that the Bcl-2 family, which includes anti-apoptotic Bcl-2 and pro-apoptotic Bax, plays a regulatory role in cell apoptosis (34). Under normal conditions, the expressions of Bcl-2 and Bax are balanced. Under abnormal conditions, however, overexpressed Bcl-2 could dissociate Bax dimers and promote the formation of complexes of Bax and Bcl-2, then inhibit cell apoptosis, and up-regulated Bax increases the number of Bax dimers and promote cell apoptosis (34). Thus, the ratio of Bcl-2/Bax is considered a predictive index in regulating cell survival and death (34,35). Our study suggested that the down-regulated Bax and up-regulated Bcl-2 were consistent with the anti-apoptotic role of etanercept in the cell model of cardiomyocyte hypertrophy. Furthermore, a previous study has proved that up-regulated Bcl-2 could attenuate low ambient temperature-induced myocardial hypertrophy *in vivo* (36). Another study also proved that Cyclovirobuxinum D alleviated cardiac hypertrophy in rats through repression of cardiac cell apoptosis (37). Taken together, the protective effect of etanercept on cardiomyocyte hypertrophy might be associated with cell apoptosis.

Furthermore, the present study found that etanercept inhibited the expressions of IL-1β and proteins in the IL-6 family, including IL-6, LIF and CT-1, in the cell model of cardiomyocyte hypertrophy. Considering that IL-1β and IL-6 family proteins possess potent pro-inflammatory actions, etanercept might exert an anti-inflammatory effect on cardiomyocyte hypertrophy. Consistently, Don et al. (38) revealed that etanercept could inhibit the systemic inflammatory response in chronic hemodialysis patients. Galarraga et al. (39) also reported that etanercept could improve arterial stiffness by inhibiting inflammatory response in rheumatoid arthritis. To further determine anti-inflammatory signaling pathway of etanercept in cardiomyocyte hypertrophy, we detected the expression of TGF-β1. Unfortunately, etanercept did not change the level of TGF-β1 in cardiomyocyte hypertrophy, which suggests that TGF-β1 might not be involved in the anti-inflammatory role of etanercept in cardiomyocyte hypertrophy. Thus, further investigation is essential to understand the signaling pathway underlying TNF-α-induced inflammatory response in cardiomyocyte hypertrophy.

In summary, etanercept may improve rat cardiomyocyte hypertrophy by inhibiting inflammatory cytokines secretion and cell apoptosis.
